# Extracorporeal Ultrasound-Guided High Intensity Focused Ultrasound: Implications from the Present Clinical Trials

**DOI:** 10.1155/2014/537260

**Published:** 2014-04-03

**Authors:** Tinghe Yu, Xiao Fu

**Affiliations:** Key Medical Laboratory of Obstetrics and Gynecology, The Second Affiliated Hospital, Chongqing Medical University, Chongqing 400010, China

## Abstract

Extracorporeal ultrasound-guided high intensity focused ultrasound (HIFU) has been clinically used for 15 years, and over 36000 cases have been reported. However, there yet lacked a consensus in the clinical values, suggesting the necessity of checking clinical findings. Clinical trials were searched and data reevaluated. HIFU was hardly performed alone; almost all present anticancer means have been applied during an HIFU treatment, and a specific regimen varied between trials; there were heterogeneity and disagreement between trials. The complexity made it difficult to distinguish the effect of HIFU. Based upon evaluable data, the efficacy of HIFU was similar to that of radio frequency, chemoembolization, chemotherapy, radiotherapy, or hormone therapy; a combined therapy did not improve the efficacy. The survival rate of HIFU plus radiotherapy was lower than that of radical surgery in liver cancers. Adverse events had no downtrend in the past years. HIFU was not a standardized procedure where the intensity and insonation mode were modified constantly throughout a treatment, limiting an evaluation from the perspective of ultrasonics. These implied that HIFU should be applied as an alternative at most occasions. The present clinical trials had defects making against the understating of HIFU.

## 1. Introduction

High intensity focused ultrasound (HIFU) can be focused on the preselected volume within the body without harming overlying tissues, thereby inducing heat and cavitation affecting the target area. HIFU treatment is guided by ultrasound or magnetic resonance image, which is even considered as the future of surgery for the noninvasive essence [1].

Extracorporeal ultrasound-guided HIFU (USgFU) has been clinically used for 15 years. This modality has been applied to cancers of liver, pancreas, and kidney in Europe and Asia and the feasibility has been demonstrated [2, 3]. HIFU has been used to manage many types of benign and malignant diseases in China and some advocates state that it is safe and effective [4, 5]. However, those data are usually published in Chinese and only few trials are released in English, which makes it difficult for scientists outside China to catch HIFU. Over 36000 cases have been reported till 2012. Theoretically, such large quantities of data provide comprehensive information. However, there still lacks a consensus in the clinical values. Indeed, the efficacy in bone tumors has been queried [6]. These suggest that the present clinical findings should be checked.

In the present study, clinical trials were searched and reevaluated and some limitations outlined. Those defects have been limiting the understating of HIFU and should be avoided in following trials. HIFU should be an alternative for a subset of patients in some disease types.

## 2. Methods

### 2.1. Searching Clinical Trials

Clinical trials were searched in the Chinese Scientific and Technical Periodicals Database and the China National Knowledge Infrastructure.

### 2.2. Statistics

Data were extracted and reevaluated. The rates were compared with the chi-square test and multitest with the bootstrap. Statistics were performed with the software SAS (SAS Inst., Cary, NC).

## 3. Results and Discussion

### 3.1. General

36454 cases were reported in 846 papers. Most trials were case-series. 66 controlled trials were used for the reevaluation for adopting the response evaluation criteria in solid tumors.

### 3.2. Therapeutic Regimens

A benign lesion was usually treated with single HIFU. Combined therapies included the use of mifepristone, iohexol, or artery embolization in uterine fibroid and finasteride in prostate hyperplasia. Benign diseases can be managed by partial ablation. An adjuvant was applied as a pre-HIFU means increasing the ablation rate via inducing the shrinkage and devasculation of a lesion, or as a post-HIFU strategy reducing the relapse [7].

HIFU was hardly performed alone in cancers. Almost all present anticancer means have been used during an HIFU treatment (Table 1). Moreover, a regimen varied between trials, even a specific strategy for a specific disease type, such as transarterial chemoembolization (TACE) and chemotherapy. TACE was actually a combined treatment (cutoff of the blood supply, arterial infusion, and systemic chemotherapy), which was a poor reference for drastic variance in the operative technique and ingredients applied [8]; thus, a clinical trial using TACE as the reference cannot provide convincing evidence to support the benefit of the candidate modality. HIFU was not a standardized procedure (the intensity and insonation mode were modified constantly throughout a treatment) [9]. The combination undoubtedly resulted in an intricacy. Thus, it was not surprising that there was disagreement between trials. The complexity made it difficult to distinguish the efficacy of HIFU and to merge data to perform a standard meta-analysis. The reason for the prevalence of TACE in an HIFU treatment remained unclear. That HIFU was performed several weeks after TACE indicated that TACE had caused cell death or irreversible damage before HIFU, suggesting HIFU an enhancer of TACE. Drugs varied in chemotherapy, even for a specific disease type. The more adjuvant a therapy required, the weaker potency the modality itself was. Multiple therapeutic strategies suggested that HIFU should be an alternative at most occasions. These demerits should be considered when designing an HIFU trial. Single therapy with confirmed clinical benefit should be set as the reference.

### 3.3. Efficacy

Most trials were case-series. The efficacy can be objectively assessed in controlled trials. Based upon evaluable controlled trials, the benefit of HIFU was similar to that of surgery or drugs in benign disease types and to radio frequency, TACE, chemotherapy, radiotherapy, or hormone therapy in cancers (Table 2). HIFU therefore should be restricted to a subgroup. A guideline should be developed to determine those cases, and the ratios of harm-benefit and cost-benefit should be assessed.

HIFU plus 3-dimensional conformal radiotherapy (3DCRT) was compared with the radical surgery in resectable liver cancer. The response rate was 97.1% versus 100% (*P* = 0.2365) but the surgery had a higher rate of complete response (70.6% versus 94.1%, *P* = 0.0083). The 1-, 2-, and 5-year survival rates were 91.2%, 85.3%, and 58.8% in the combination and 97.1%, 94.1%, and 82.4% in surgery (*P* = 0.2923, 0.2244, and 0.0314) [21]. The clinical benefit of 3DCRT has been confirmed [22]. HIFU alone therefore may produce a poorer outcome and should be curtailed in patients with a surgical opportunity.

It was difficult to outline the efficacy of a combined therapy because of the disagreement between trials and the variance of a specific therapeutic regimen between trials. Several therapeutic strategies can be evaluated. HIFU was combined with 3DCRT to treat advanced cancer; response rates were 37.5% and 22.7% (*P* = 0.2739) in the combination and 3DCRT, respectively [23]. In prostate cancer, the 5-year survival rate of HIFU plus emasculation was similar to that of emasculation (83.3% versus 66.7%, *P* = 0.4430) [20]. Data can be merged in pancreatic cancer; the response rates were 30.8% in HIFU combined with chemotherapy (gemcitabine plus cisplatin) and 27.1% in chemotherapy (*P* = 0.6846); HIFU plus 3DCRT did not improve the response rate compared with 3DCRT (38.3% versus 24.6%, *P* = 0.1025). These findings did not demonstrate that HIFU enhanced other regimens. A combined therapy, therefore, should not be recommended at most occasions. HIFU directly ablated tissues, but drugs and radiation deactivated cells via a series of intracellular processes. Cancer type and drug were the determents of the interaction, and HIFU cavitation can decrease the potency of a drug [24, 25]. Thus, only a specific regimen can be introduced during HIFU for a specific case. The efficacy of a combined therapy including the sequence effect should be explored. The improper coadministration of an adjuvant led to unexpected events—drug or radiation induced predamages to noncancerous tissues thereby increasing the risk of untoward effects due to HIFU [26].

### 3.4. Heterogeneity

HIFU plus 3DCRT was compared with 3DCRT in the management of retroperitoneal metastasis in 2 trials. Response rates of the combination versus 3DCRT were 48.0% versus 28.0% (*P* = 0.1434) and 86.4% versus 60.0% (*P* = 0.0523), respectively [27, 28]. Noticeably, the evaluation with merged data indicated that the combination improved the efficacy (66.0% versus 42.2%, *P* = 0.0217).

The illogicality demonstrated the heterogeneity of present clinical trials. A conventional meta-analysis, therefore, cannot be applied to HIFU. Aforementioned event may be only the tip of an iceberg, considering the diversity of therapeutic strategies. The heterogeneity was related to the cases involved. HIFU was usually applied to patients with an advanced cancer or failure to other treatments (i.e., inhomogeneity between individuals), and the insonation mode needs to be constantly modified throughout a treatment (i.e., inherent inhomogeneity) [9]. Thus, the quality of clinical trials of HIFU was unsatisfactory and it was difficult to eliminate the heterogeneity. The heterogeneity made it difficult to generalize the clinical benefit of HIFU despite 15 years of clinical experience and >36000 reported cases.

The heterogeneity decreased the feasibility of a randomized controlled trial (RCT). Indeed, several recent papers published in English cannot be classed as RCT [29, 30]. This limited the understanding of clinical benefit of HIFU. To debase the heterogeneity was a great challenge for an HIFU clinical trial.

### 3.5. Adverse Events

Adverse events were described detailedly in few controlled trials. In uterine fibroid, the rates of adverse events were 75.0% in HIFU and 9.5% in surgery (*P* < 0.0001) and were 36.0% in radio frequency and 16.0% in HIFU (*P* = 0.0213) [10, 31]. The rate in HIFU was lower than that in chemotherapy (20.0% versus 55.0%, *P* = 0.0203) in pancreatic cancer [18]. The present data cannot support the allegation that HIFU had fewer untoward effects. Adverse events can be evaluated in RCT. The combined regimen made it difficult to identify HIFU-related toxicities, so there lacked consentaneous data yet. This should be considered in following trials.

**Figure 1:**
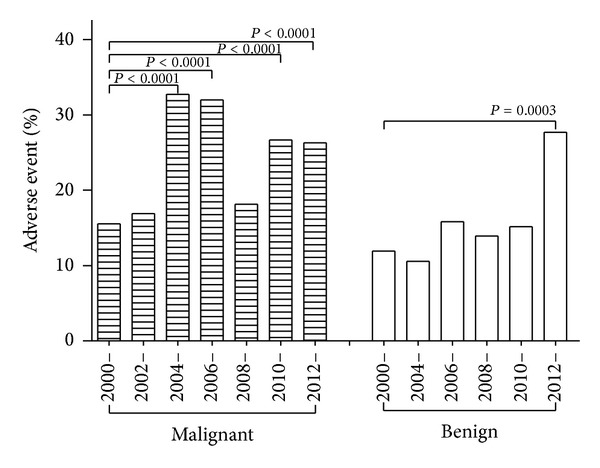
Chronological analysis of the rate of adverse events of HIFU during 2000–2012. There was no downtrend in either malignant or benign disease types.

Adverse events in 18596 reported cases during 2000–2012 were generalized. There was no downtrend in either benign or malignant disease types (Figure 1). A previous trial indicated that the rate of adverse events varied between disease types (up to 280%), and the location of a lesion and the HIFU device were the determinants of side effects; severe toxicities, such as lung embolism, occlusion of the superior mesenteric artery, intrahepatic metastasis, and tumor rupture, occurred; some (metastasis and artery occlusion) were inconsistent with the verdicts established in preclinical studies [26]. Empirically, only a fraction of undesired events were reported [32]. The actual incidence of untoward events will be much higher. Theoretically, adverse events decreased with the development of HIFU devices and the accumulation of clinical experiences. The assumption was not supported with the present data, suggesting that HIFU was still at an early stage.

It was believed that tissue response was monitored in real time during a USgFU treatment, suggesting fewer adverse events. Indeed, this was not the fact. Because diagnostic ultrasound must be suspended during the release of therapeutic ultrasound, there was a short delay (milliseconds) to acquire tissue information [9]. Consequently, only the insonation outcome was viewed but the tissue changes during insonation were actually invisible, which was a bug of USgFU. Ultrasound with a frequency of 1–5 MHz corresponded to a wavelength of 1.5–0.3 mm (far larger than the cell diameter). The therapeutic precision of HIFU therefore was lower than that of radiation (wavelength of <10 nm), which was decreased further by the nonlinear behavior of ultrasound in tissues. HIFU may destruct tissues outside the focus, leading to untoward damages. The only approach to improve the safety was to understand the tissue-ultrasound interactions. The low precision may be one of the reasons that the survival time in radiotherapy was longer than that in HIFU in pancreatic cancer [19].

### 3.6. Operative Manner and Parameters

Only the acoustic power was described in most trials, but the intensity, exposure duration, and insonation mode were not described. HIFU utilized heat and cavitation—the intensity was the leading physical parameter for both therapeutic effect and toxicity [9]. Thus, the present clinical data cannot be generalized to evaluate the benefits from the perspective of ultrasonics. HIFU was not a standardized procedure; the insonation manner needed constant modifications throughout a treatment according to tissue responses—a process depending on the experience of physicians. Thus, HIFU was at a high risk of misplay; underdose-insonation reduced the efficacy and overdose led to unexpected tissue damages. The diversity of insonation manner made it difficult to outline the “dose-effect” and “dose-toxicity” relationships of HIFU therapy, even in a specific disease type. As an example, the required ultrasonic energy to necrotize 1 cm^3^ of human uterine fibroid had a 77× disparity in trials [9].

As a complex medical device, to operate USgFU properly was not easy. USgFU was the integration of diagnostic and therapeutic ultrasound (i.e., relating to both medicine and physics). The manipulation of a USgFU device placed high demands on the operator. Too many man-reliant processes were a critical root of human blunders [32]. This device therefore was in connection with a high risk of improper manipulation leading to poor therapeutic outcomes and side effects. However, no concerns have been given over this plight.

### 3.7. Summary

The present clinical data did not provide convincing data to support the alleged advantage of HIFU, including the therapeutic efficacy and adverse events. HIFU should be applied as an alternative for a subset of patients, in most disease types. The present clinical trials had flaws thereby limiting the understating of clinical benefits of HIFU. These should be considered in following trials to develop this emerging therapeutic modality.

## Figures and Tables

**Table 1:** List of the therapeutic strategies applied during HIFU for cancers.

Disease and treatment	Regimen
Liver	
TACE	ADM
	BLM
	DDP
	5-FU + ADM
	5-FU + DDP
	5-FU + EPI
	5-FU + GEM
	GEM + Oxa
	5-FU + ADM + CBP
	5-FU + ADM + MMC
	5-FU + CBP + MMC
	5-FU + DDP + EPI
	5-FU + DDP + MMC
	5-FU + EPI + Oxa
	ADM + FUDR + MMC
	FUDR + Oxa + THP
	5-FU + (ADM/THP) + (DDP/Oxa)
	5-FU + AMD + DDP + MMC
	5-FU + DDP + EPI + MMC
	5-FU + MMC + THP + CF
	5-FU, (DDP/Oxa), EPI, GEM
	5-FU, DDP, EPI, HCPT, MMC
Chemotherapy	Capecitabine
	5-FU + CBP
	DDP + GEM
	5-FU + ADR + DDP
	5-FU + CTX + VCR
	ADR + CTX + DDP
	5-FU + Oxa + CF
Radiotherapy	3DCRT
	Stereotactic
Biotherapy	Thymosin *α*1
	Tumor necrosis factor
	DCCIK
Surgery	
Ethanol	
Others	Chlorin e6
	Thalidomide

Pancreas	
Chemotherapy	5-FU
	Capecitabine
	DDP
	GEM
	5-FU + CF
	5-FU + GEM
	GEM + Capecitabine
	GEM + DDP
	GEM + Oxa
	5-FU + GEM + CF
Arterial infusion	GEM
	5-FU + GEM
	5-FU + DDP + EPI
	5-FU + GEM + Oxa
	DDP + GEM + Interferon
	5-FU + EPI + MMC + CF
Radiotherapy	3DCRT
Celiac ganglia destruction	
Biotherapy	Mycobacterium phlei F.U.36
	Octreotide

Ovary	
Chemotherapy	BLM + DDP + VCR
	CTX + DDP + VCR
Radiotherapy	

Uterine cervix	
Chemotherapy	5-FU + DDP + CF
	CTX + BLM + DDP
	5-FU + DDP + PTX + CF
Radiotherapy	

Vagina	
Radiotherapy	

Bone	
Arterial infusion	DDP
	ADM, DDP, MTX, IFO
Chemotherapy	ADM + DDP
	HDMTX + VCR
	ADM + MTX + IFO
	ADM, DDP, MTX, IFO
Radiotherapy	

Breast	
Chemotherapy	PTX + ADM
	PTX + EPI
	5-FU + ADM + CTX
	5-FU + CTX + EPI
	5-FU + CTX + MTX
	CTX + EPI + Tegafur
Hormone therapy	Tamoxifen
Radiotherapy	
Endoscopic axillary node dissection	

Soft tissues	
Chemotherapy	ADM + DDP
	DDP + IFO
	CBP + VP-16
	ADM + DTIC + IFO
	CBP + EPI + VCR
Arterial infusion	DDP
	ADM + DTIC + IFO
Radiotherapy	
Surgery	

Retroperitoneal lesions	
Radiotherapy	3DCRT
Chemotherapy	N/A
Surgery	

Esophagus	
Chemotherapy	DDP + PTX
	5-FU + DDP + CF

Stomach	
Chemotherapy	5-FU + DDP + PTX
	5-FU + Oxa + CF

Colorectum	
Chemotherapy	5-FU + ADM + MTX
	5-FU + Oxa + CF
Radiotherapy	

Kidney	
Biotherapy	Interferon

Prostate	
Hormone therapy	Orchidectomy
	Flutamide/bicalutamide
	Leuprorelin/goserelin
Radiotherapy	^ 125^I
	External beam
Transurethral resection	

Bladder	
Infusion	ADM
	HCPT
	MMC
Radiotherapy	

5-FU: 5-fluorouracil; ADM: adriamycin; BLM: bleomycin; CBP: carboplatin; CF: calcium folinate; CTX: cyclophosphamide; DDP: cisplatin; DTIC: dacarbazine; EPI: epirubicin; FUDR: floxuridine; GEM: gemcitabine; HCPT: hydroxycamptothecin; IFO: ifosfamide; MMC: mitomycin C; MTX: methotrexate; Oxa: oxaliplatin; PTX: paclitaxel; THP: pirarubicin, VCR: vincristine; VP-16: etoposide.

**Table 2:** The efficacy of HIFU in controlled trials.

Disease and treatment (number of cases)	Response	Reference
Uterine fibroid		
HIFU (72) Myomectomy (74)	87.5% versus 94.6% (*P* = 0.1282)	[10]
HIFU (49) Mifepristone (53)	Tumor shrinkage 95.9% versus 90.6% (*P* = 0.2770) Symptom relief 93.9% versus 96.2% (*P* = 0.5824)	[11]
Mifepristone (20) HIFU (20) HIFU + mifepristone (20)	85.0% versus 90.0% versus 95.0% (*P* = 0.5606)	[12]

Ectopic pregnancy		
HIFU (20) Mifepristone + methotrexate (20)	80.0% versus 85.0% (*P* = 0.6769)	[13]

Chyluria		
HIFU (25) Lymphatic disconnection (30)	84.0% versus 83.3% (*P* = 0.9469) Relapse 14.3% versus 16.0% (*P* = 0.8717)	[14]

Liver cancer		
HIFU (20) Radio frequency (20)	3-, 6-, 9-, and 12-month survival 80.0%, 61.1%, 42.9%, and 33.3% versus 85.0%, 58.8%, 46.7%, and 36.4% (*P* = 0.6769, 1.00, 1.00, 1.00)	[15]
HIFU (30) TACE (30)	0.5-, 1-, and 2-year survival 83.3%, 63.3%, and 40.0% versus 66.7%, 43.3%, and 23.3% (*P* = 0.1331, 0.1192, 0.1634)	[16]
HIFU (40) *γ*-knife (38)	Early response 20.0% versus 39.5% (*P* = 0.0580) PVTT 52.5% versus 47.4% (*P* = 0.6504) MST 10 versus 11 months* (NS)	[17]

Pancreas cancer		
HIFU (20) Chemotherapy (20)	Early response 50.0% versus 30.0% (*P* = 0.1949) 6-month survival 70.0% versus 50.0% (*P* = 0.1949)	[18]
HIFU + 3DCRT (22) 3DCRT (29) HIFU (22)	Early response 63.6% versus 44.8% versus 40.9% (*P* = 0.2611) 0.5-, 1-, and 2-year survival 95.5%, 59.1%, and 50.0% versus 93.1%, 41.4%, and 24.1% versus 95.5%, 40.9%, and 22.7% (*P* = 0.9127, 0.3704, 0.0891) MST 17.6 versus 12.4 versus 12.3 months	[19]

Prostate cancer		
HIFU + emasculation (21) Emasculation (19)	5-year survival 83.3% versus 66.7% (*P* = 0.4430) Bone metastasis 27.8% versus 50.0% (*P* = 0.3053)	[20]

PVTT: portal vein tumor thrombosis; MST: median survival time.

*The raw data were not described.
